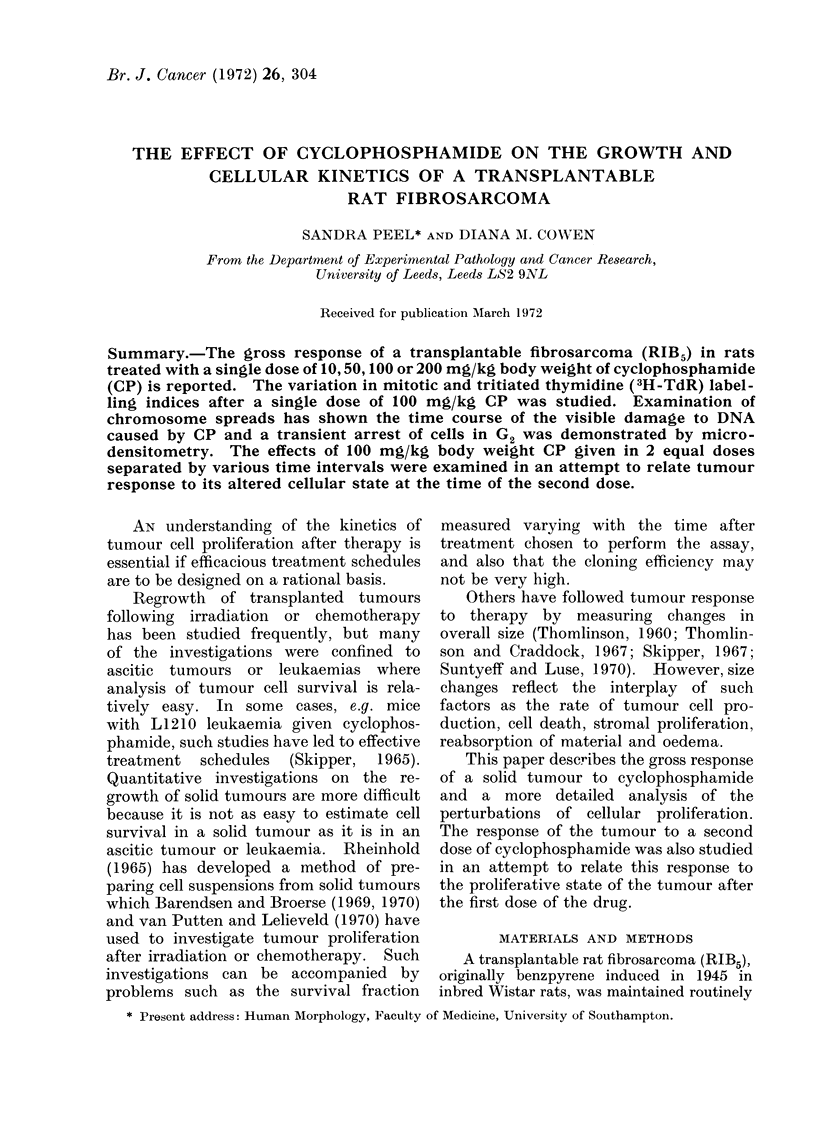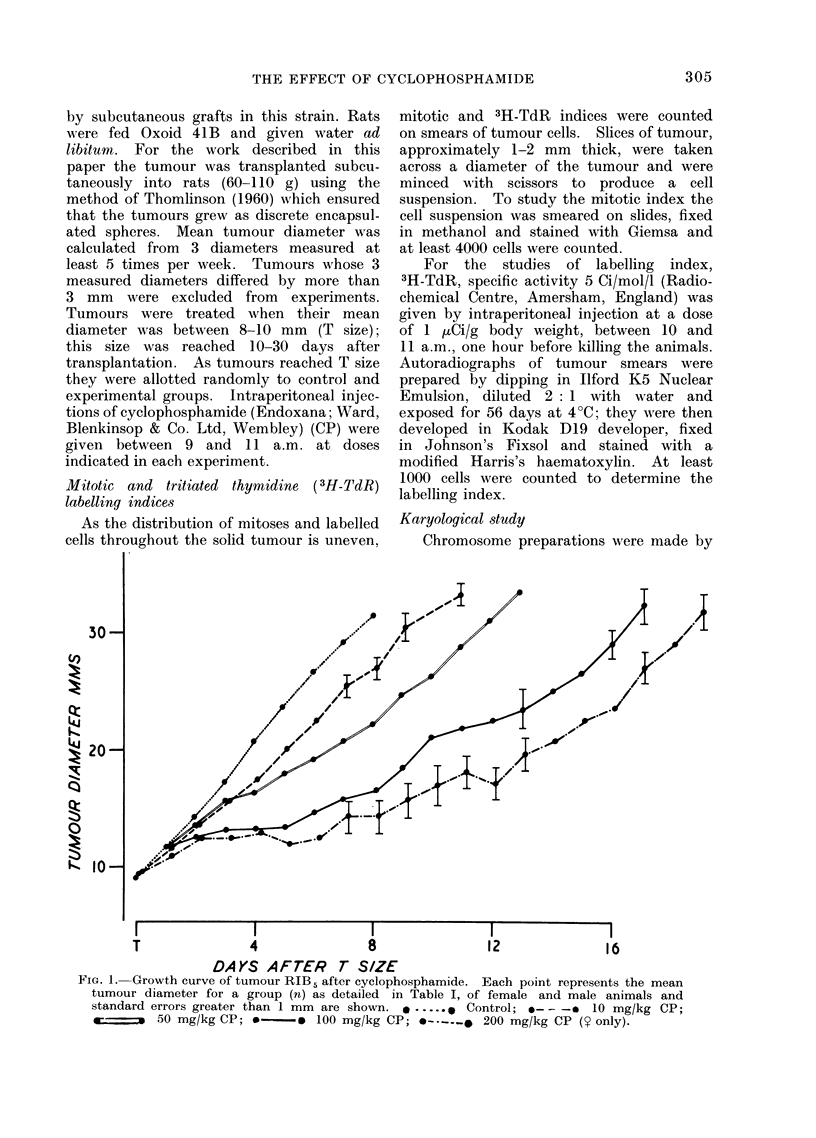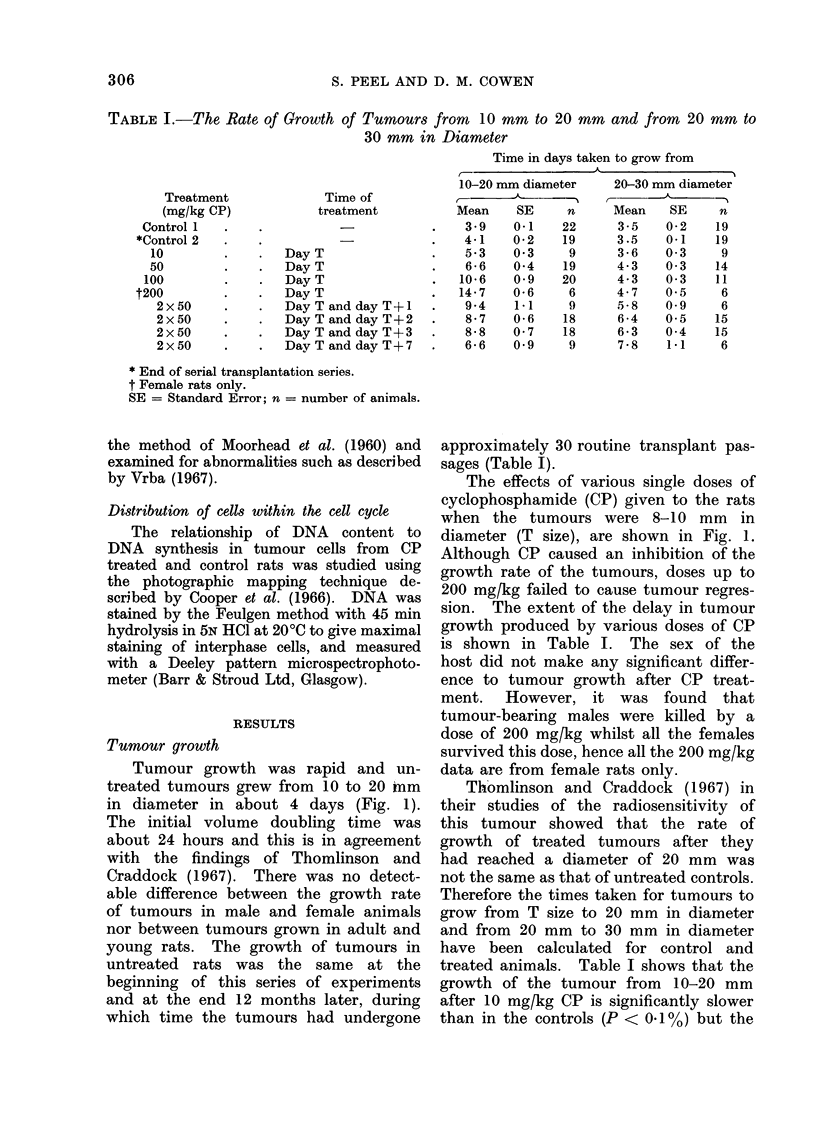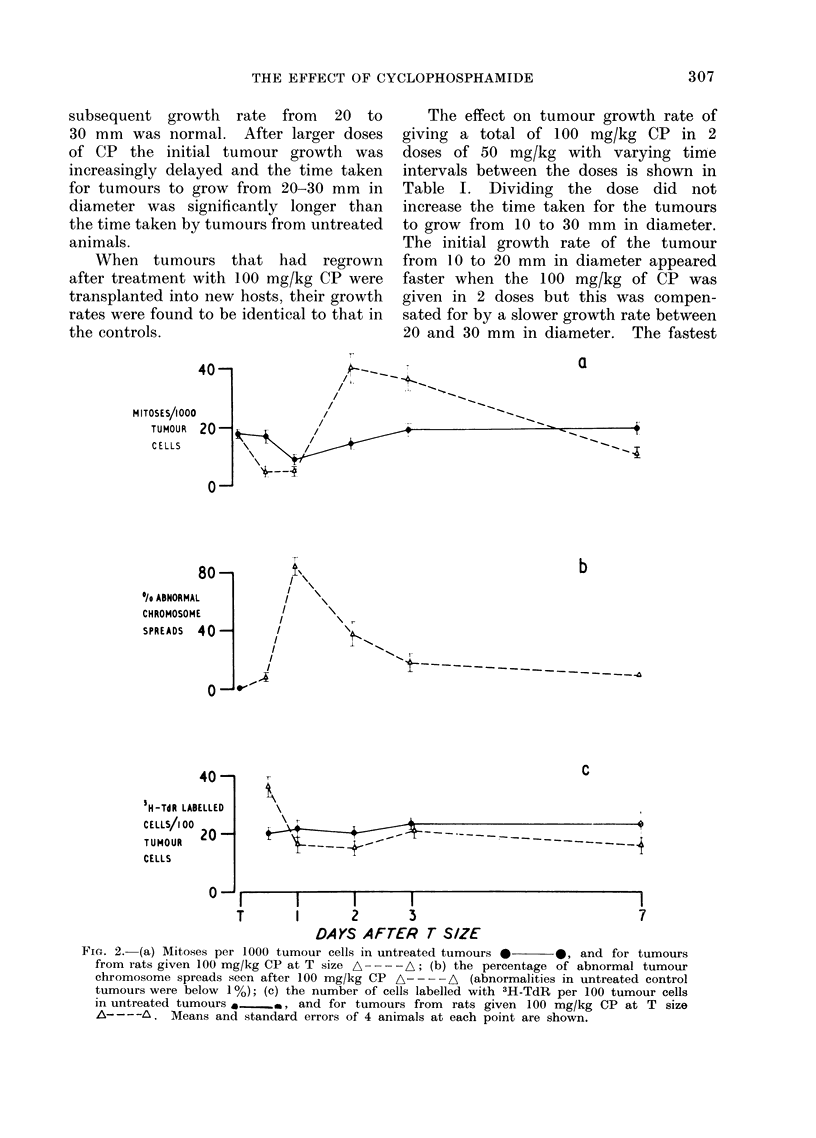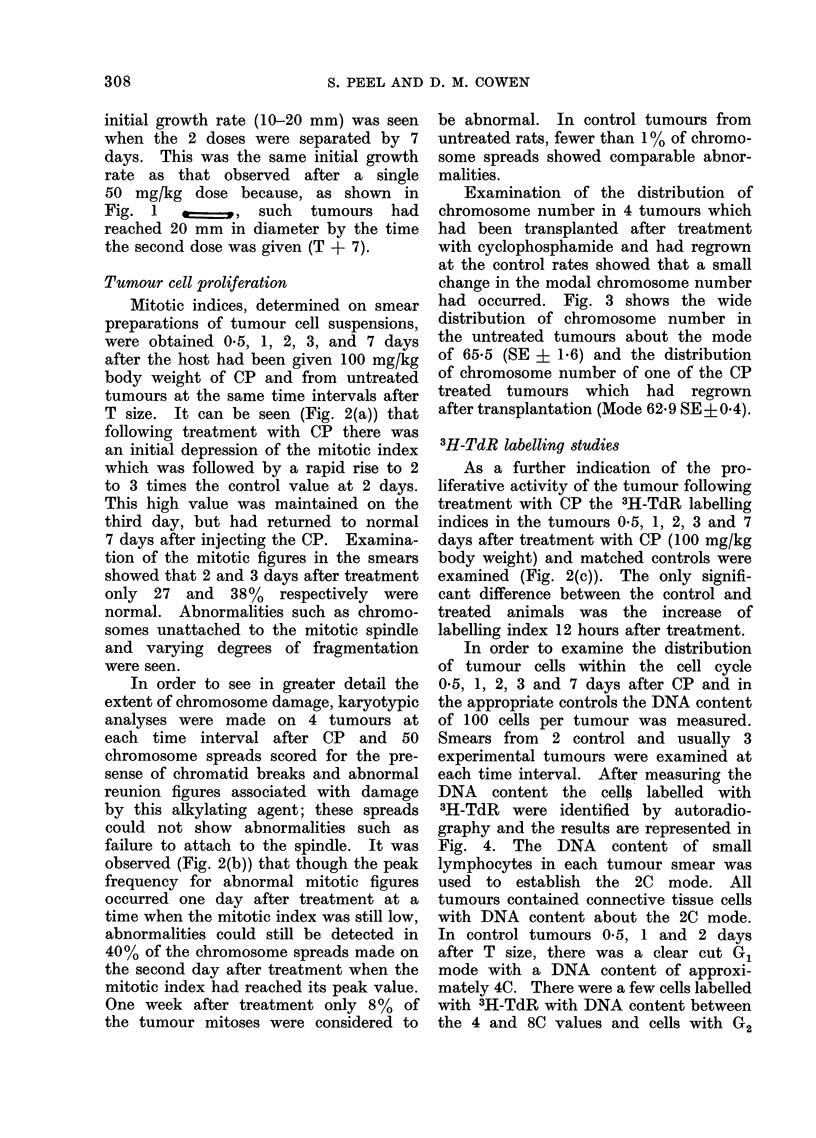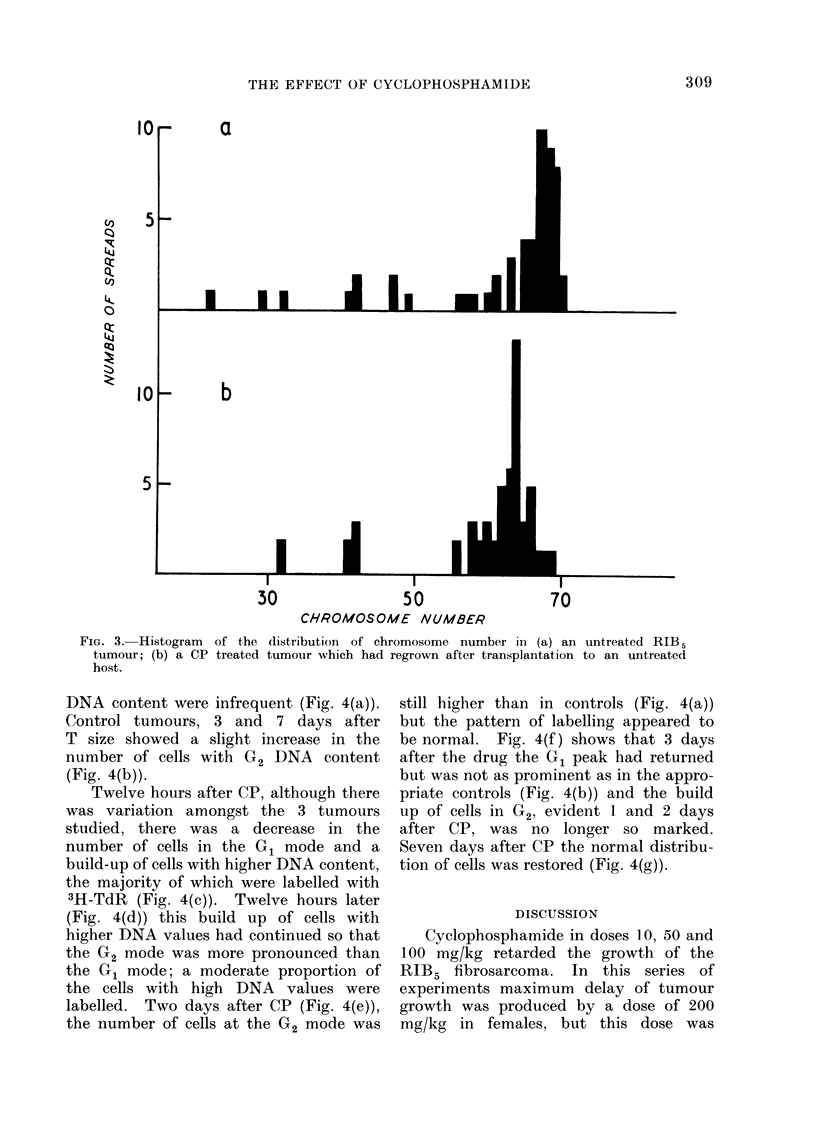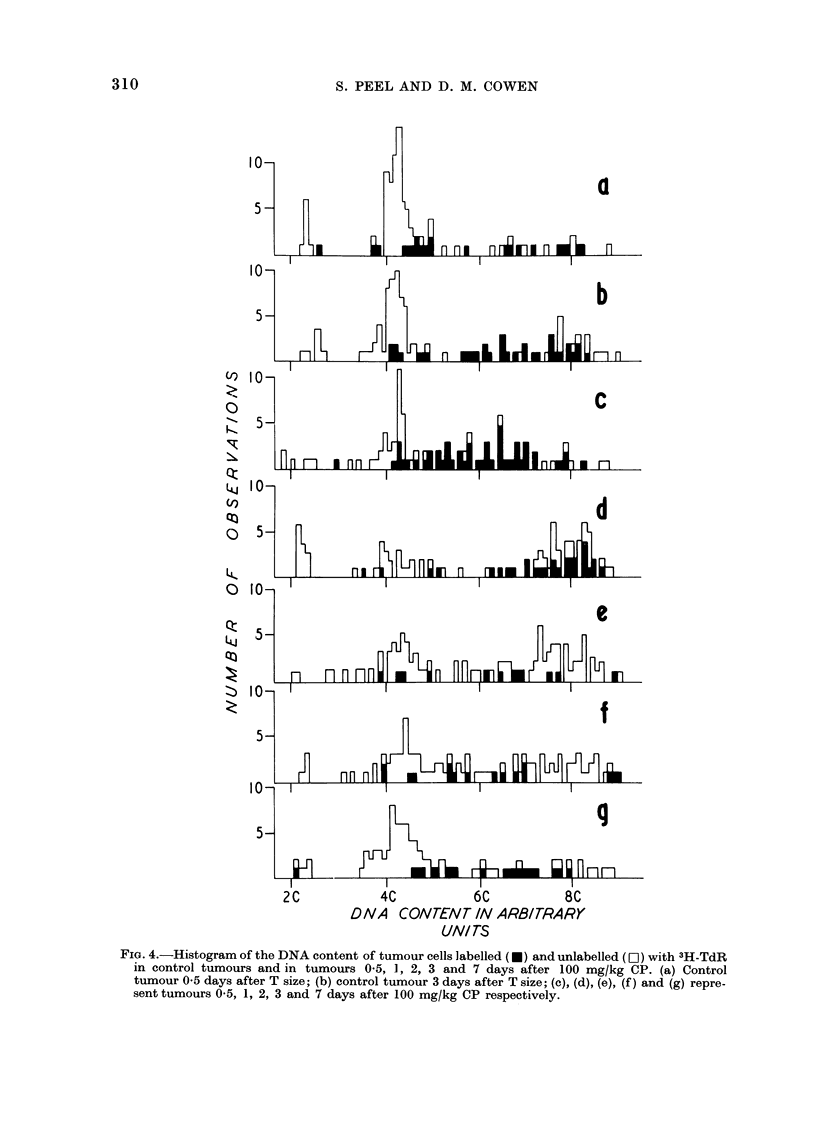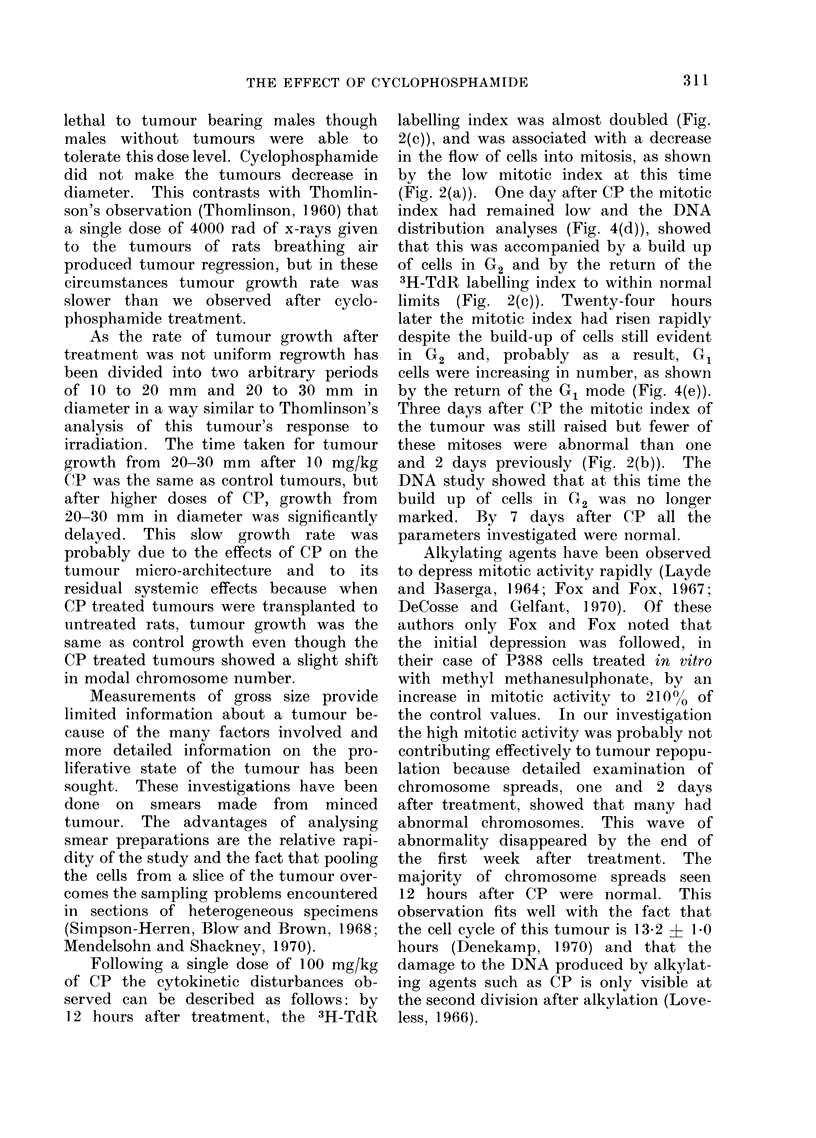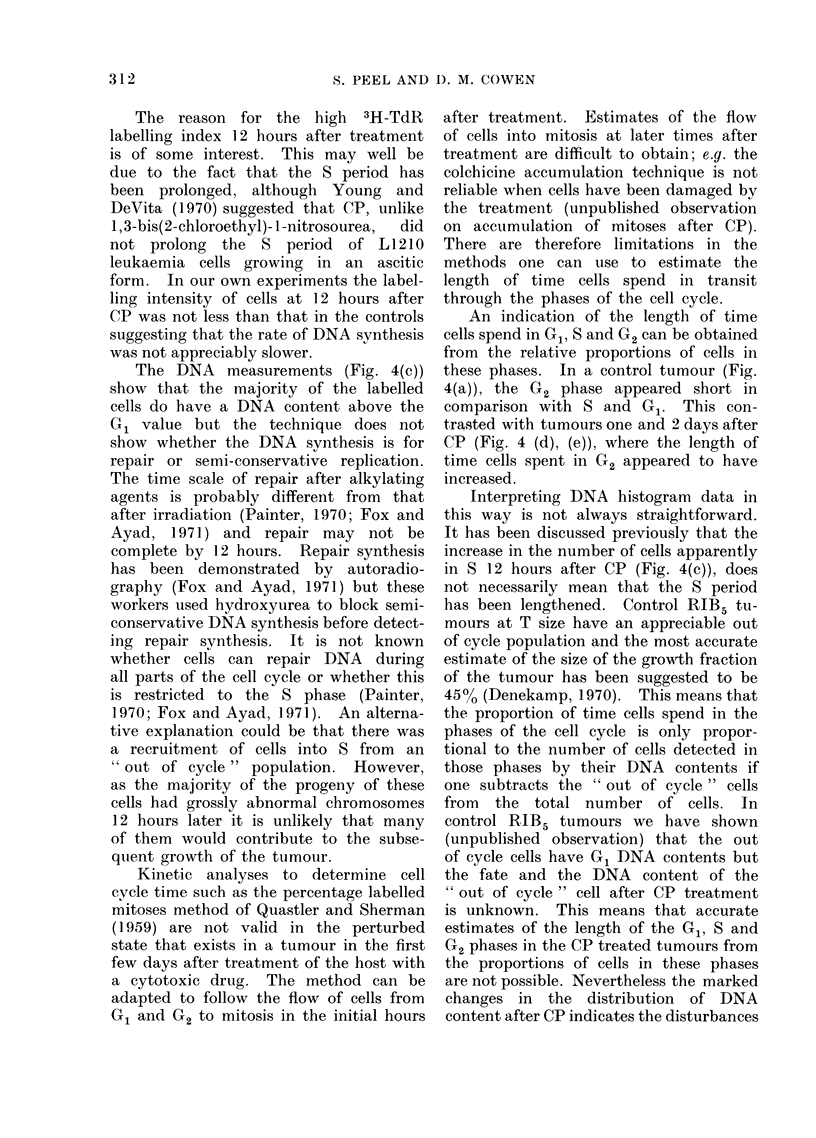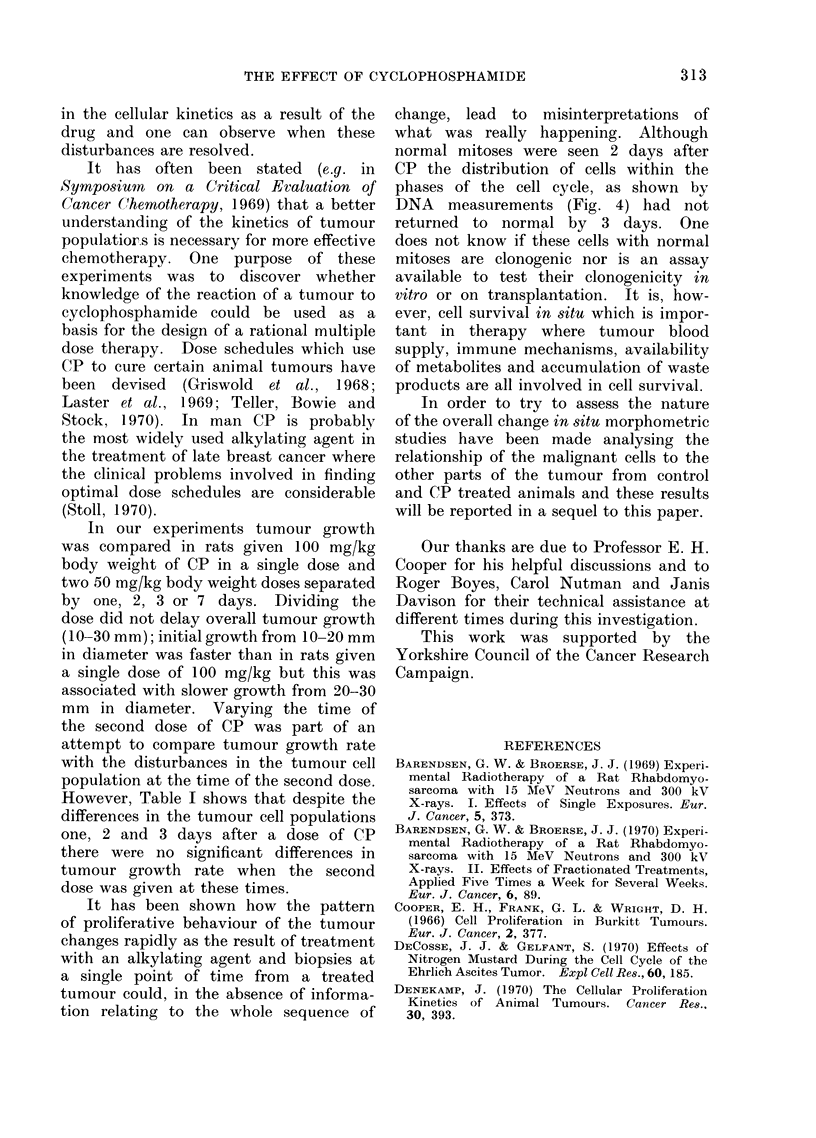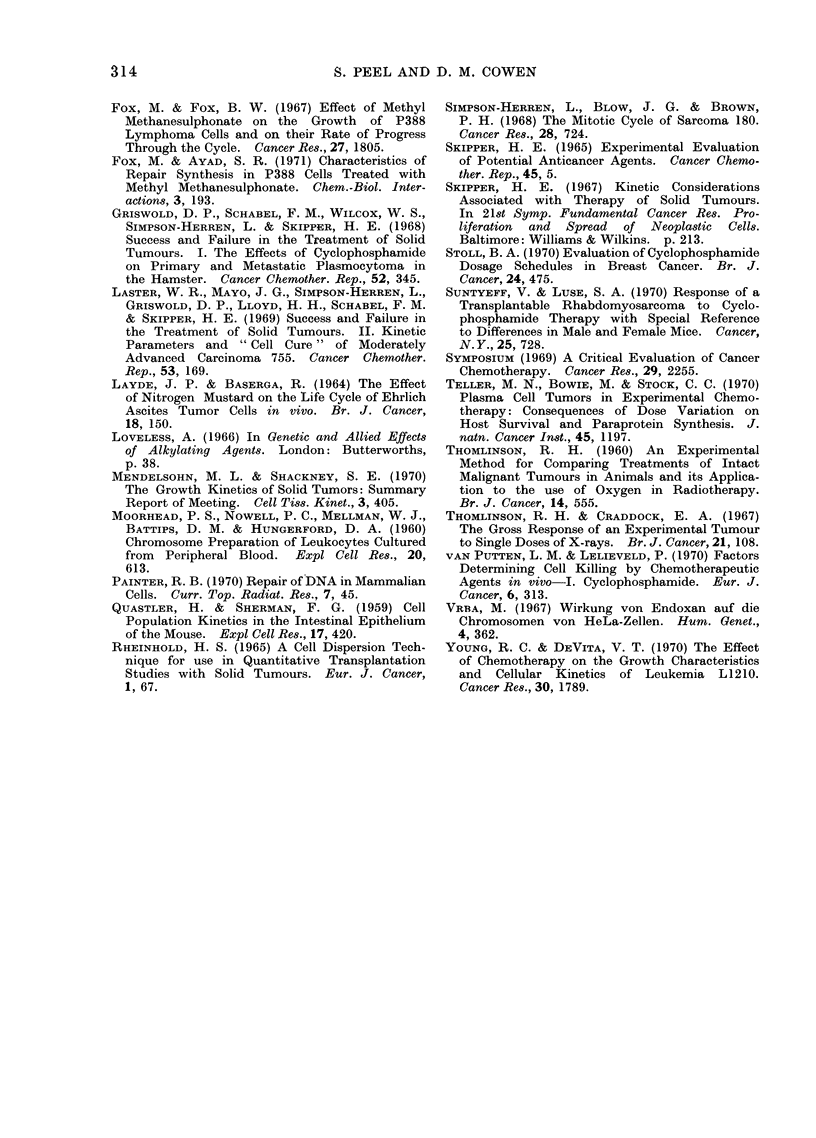# The Effect of Cyclophosphamide on the Growth and Cellular Kinetics of a Transplantable Rat Fibrosarcoma

**DOI:** 10.1038/bjc.1972.41

**Published:** 1972-08

**Authors:** Sandra Peel, Diana M. Cowen

## Abstract

The gross response of a transplantable fibrosarcoma (RIB_5_) in rats treated with a single dose of 10, 50, 100 or 200 mg/kg body weight of cyclophosphamide (CP) is reported. The variation in mitotic and tritiated thymidine (^3^H-TdR) labelling indices after a single dose of 100 mg/kg CP was studied. Examination of chromosome spreads has shown the time course of the visible damage to DNA caused by CP and a transient arrest of cells in G_2_ was demonstrated by microdensitometry. The effects of 100 mg/kg body weight CP given in 2 equal doses separated by various time intervals were examined in an attempt to relate tumour response to its altered cellular state at the time of the second dose.


*Br. J. Cancer* (1972) **26**, 304

### THE EFFECT OF CYCLOPHOSPHAMIDE ON THE GROWTH AND CELLULAR KINETICS OF A TRANSPLANTABLE RAT FIBROSARCOMA

SANDRA PEEL* AND DIANA M. COWEN

*From the Department of Experimental Pathology and Cancer Research, University of Leeds, Leeds LS2 9NL*

Received for publication March 1972

**Summary.—The gross response of a transplantable fibrosarcoma ($RIB_5$) in rats treated with a single dose of 10, 50, 100 or 200 mg/kg body weight of cyclophosphamide (CP) is reported. The variation in mitotic and tritiated thymidine ($^3$H-TdR) labelling indices after a single dose of 100 mg/kg CP was studied. Examination of chromosome spreads has shown the time course of the visible damage to DNA caused by CP and a transient arrest of cells in $G_2$ was demonstrated by microdensitometry. The effects of 100 mg/kg body weight CP given in 2 equal doses separated by various time intervals were examined in an attempt to relate tumour response to its altered cellular state at the time of the second dose.**

An understanding of the kinetics of tumour cell proliferation after therapy is essential if efficacious treatment schedules are to be designed on a rational basis.

Regrowth of transplanted tumours following irradiation or chemotherapy has been studied frequently, but many of the investigations were confined to ascitic tumours or leukaemias where analysis of tumour cell survival is relatively easy. In some cases, *e.g.* mice with L1210 leukaemia given cyclophosphamide, such studies have led to effective treatment schedules (Skipper, 1965). Quantitative investigations on the regrowth of solid tumours are more difficult because it is not as easy to estimate cell survival in a solid tumour as it is in an ascitic tumour or leukaemia. Rheinhold (1965) has developed a method of preparing cell suspensions from solid tumours which Barendsen and Broerse (1969, 1970) and van Putten and Lelieveld (1970) have used to investigate tumour proliferation after irradiation or chemotherapy. Such investigations can be accompanied by problems such as the survival fraction measured varying with the time after treatment chosen to perform the assay, and also that the cloning efficiency may not be very high.

Others have followed tumour response to therapy by measuring changes in overall size (Thomlinson, 1960; Thomlinson and Craddock, 1967; Skipper, 1967; Suntyeff and Luse, 1970). However, size changes reflect the interplay of such factors as the rate of tumour cell production, cell death, stromal proliferation, reabsorption of material and oedema.

This paper describes the gross response of a solid tumour to cyclophosphamide and a more detailed analysis of the perturbations of cellular proliferation. The response of the tumour to a second dose of cyclophosphamide was also studied in an attempt to relate this response to the proliferative state of the tumour after the first dose of the drug.

### MATERIALS AND METHODS

A transplantable rat fibrosarcoma ($RIB_5$), originally benzpyrene induced in 1945 in inbred Wistar rats, was maintained routinely

* Present address: Human Morphology, Faculty of Medicine, University of Southampton.

by subcutaneous grafts in this strain. Rats were fed Oxoid 41B and given water *ad libitum*. For the work described in this paper the tumour was transplanted subcutaneously into rats (60–110 g) using the method of Thomlinson (1960) which ensured that the tumours grew as discrete encapsulated spheres. Mean tumour diameter was calculated from 3 diameters measured at least 5 times per week. Tumours whose 3 measured diameters differed by more than 3 mm were excluded from experiments. Tumours were treated when their mean diameter was between 8–10 mm (T size); this size was reached 10–30 days after transplantation. As tumours reached T size they were allotted randomly to control and experimental groups. Intraperitoneal injections of cyclophosphamide (Endoxana; Ward, Blenkinsop & Co. Ltd, Wembley) (CP) were given between 9 and 11 a.m. at doses indicated in each experiment.

*Mitotic and tritiated thymidine ($^3$H-TdR) labelling indices*

As the distribution of mitoses and labelled cells throughout the solid tumour is uneven, mitotic and $^3$H-TdR indices were counted on smears of tumour cells. Slices of tumour, approximately 1–2 mm thick, were taken across a diameter of the tumour and were minced with scissors to produce a cell suspension. To study the mitotic index the cell suspension was smeared on slides, fixed in methanol and stained with Giemsa and at least 4000 cells were counted.

For the studies of labelling index, $^3$H-TdR, specific activity 5 Ci/mol/l (Radiochemical Centre, Amersham, England) was given by intraperitoneal injection at a dose of 1 $\mu$Ci/g body weight, between 10 and 11 a.m., one hour before killing the animals. Autoradiographs of tumour smears were prepared by dipping in Ilford K5 Nuclear Emulsion, diluted 2 : 1 with water and exposed for 56 days at 4°C; they were then developed in Kodak D19 developer, fixed in Johnson's Fixsol and stained with a modified Harris's haematoxylin. At least 1000 cells were counted to determine the labelling index.

*Karyological study*

Chromosome preparations were made by

FIG. 1.—Growth curve of tumour RIB$_5$ after cyclophosphamide. Each point represents the mean tumour diameter for a group (*n*) as detailed in Table I, of female and male animals and standard errors greater than 1 mm are shown. •·····• Control; •– – –• 10 mg/kg CP; ═══ 50 mg/kg CP; •——• 100 mg/kg CP; •–·–·–• 200 mg/kg CP (♀ only).

TABLE I.—*The Rate of Growth of Tumours from 10 mm to 20 mm and from 20 mm to 30 mm in Diameter*

| Treatment (mg/kg CP) | Time of treatment | Time in days taken to grow from: 10–20 mm diameter Mean | SE | n | 20–30 mm diameter Mean | SE | n |
|---|---|---|---|---|---|---|---|
| Control 1 | — | 3·9 | 0·1 | 22 | 3·5 | 0·2 | 19 |
| *Control 2 | — | 4·1 | 0·2 | 19 | 3.5 | 0·1 | 19 |
| 10 | Day T | 5·3 | 0·3 | 9 | 3·6 | 0·3 | 9 |
| 50 | Day T | 6·6 | 0·4 | 19 | 4·3 | 0·3 | 14 |
| 100 | Day T | 10·6 | 0·9 | 20 | 4·3 | 0·3 | 11 |
| †200 | Day T | 14·7 | 0·6 | 6 | 4·7 | 0·5 | 6 |
| 2×50 | Day T and day T+1 | 9·4 | 1·1 | 9 | 5·8 | 0·9 | 6 |
| 2×50 | Day T and day T+2 | 8·7 | 0·6 | 18 | 6·4 | 0·5 | 15 |
| 2×50 | Day T and day T+3 | 8·8 | 0·7 | 18 | 6·3 | 0·4 | 15 |
| 2×50 | Day T and day T+7 | 6·6 | 0·9 | 9 | 7·8 | 1·1 | 6 |

* End of serial transplantation series.
† Female rats only.
SE = Standard Error; *n* = number of animals.

the method of Moorhead *et al.* (1960) and examined for abnormalities such as described by Vrba (1967).

*Distribution of cells within the cell cycle*

The relationship of DNA content to DNA synthesis in tumour cells from CP treated and control rats was studied using the photographic mapping technique described by Cooper *et al.* (1966). DNA was stained by the Feulgen method with 45 min hydrolysis in 5N HCl at 20°C to give maximal staining of interphase cells, and measured with a Deeley pattern microspectrophotometer (Barr & Stroud Ltd, Glasgow).

### RESULTS

*Tumour growth*

Tumour growth was rapid and untreated tumours grew from 10 to 20 mm in diameter in about 4 days (Fig. 1). The initial volume doubling time was about 24 hours and this is in agreement with the findings of Thomlinson and Craddock (1967). There was no detectable difference between the growth rate of tumours in male and female animals nor between tumours grown in adult and young rats. The growth of tumours in untreated rats was the same at the beginning of this series of experiments and at the end 12 months later, during which time the tumours had undergone approximately 30 routine transplant passages (Table I).

The effects of various single doses of cyclophosphamide (CP) given to the rats when the tumours were 8–10 mm in diameter (T size), are shown in Fig. 1. Although CP caused an inhibition of the growth rate of the tumours, doses up to 200 mg/kg failed to cause tumour regression. The extent of the delay in tumour growth produced by various doses of CP is shown in Table I. The sex of the host did not make any significant difference to tumour growth after CP treatment. However, it was found that tumour-bearing males were killed by a dose of 200 mg/kg whilst all the females survived this dose, hence all the 200 mg/kg data are from female rats only.

Thomlinson and Craddock (1967) in their studies of the radiosensitivity of this tumour showed that the rate of growth of treated tumours after they had reached a diameter of 20 mm was not the same as that of untreated controls. Therefore the times taken for tumours to grow from T size to 20 mm in diameter and from 20 mm to 30 mm in diameter have been calculated for control and treated animals. Table I shows that the growth of the tumour from 10–20 mm after 10 mg/kg CP is significantly slower than in the controls ($P < 0{\cdot}1\%$) but the subsequent growth rate from 20 to 30 mm was normal. After larger doses of CP the initial tumour growth was increasingly delayed and the time taken for tumours to grow from 20–30 mm in diameter was significantly longer than the time taken by tumours from untreated animals.

When tumours that had regrown after treatment with 100 mg/kg CP were transplanted into new hosts, their growth rates were found to be identical to that in the controls.

The effect on tumour growth rate of giving a total of 100 mg/kg CP in 2 doses of 50 mg/kg with varying time intervals between the doses is shown in Table I. Dividing the dose did not increase the time taken for the tumours to grow from 10 to 30 mm in diameter. The initial growth rate of the tumour from 10 to 20 mm in diameter appeared faster when the 100 mg/kg of CP was given in 2 doses but this was compensated for by a slower growth rate between 20 and 30 mm in diameter. The fastest

FIG. 2.—(a) Mitoses per 1000 tumour cells in untreated tumours ●——●, and for tumours from rats given 100 mg/kg CP at T size △– – – –△; (b) the percentage of abnormal tumour chromosome spreads seen after 100 mg/kg CP △– – – –△ (abnormalities in untreated control tumours were below 1%); (c) the number of cells labelled with $^3$H-TdR per 100 tumour cells in untreated tumours ●——●, and for tumours from rats given 100 mg/kg CP at T size △– – – –△. Means and standard errors of 4 animals at each point are shown.

initial growth rate (10–20 mm) was seen when the 2 doses were separated by 7 days. This was the same initial growth rate as that observed after a single 50 mg/kg dose because, as shown in Fig. 1 , such tumours had reached 20 mm in diameter by the time the second dose was given (T + 7).

*Tumour cell proliferation*

Mitotic indices, determined on smear preparations of tumour cell suspensions, were obtained 0·5, 1, 2, 3, and 7 days after the host had been given 100 mg/kg body weight of CP and from untreated tumours at the same time intervals after T size. It can be seen (Fig. 2(a)) that following treatment with CP there was an initial depression of the mitotic index which was followed by a rapid rise to 2 to 3 times the control value at 2 days. This high value was maintained on the third day, but had returned to normal 7 days after injecting the CP. Examination of the mitotic figures in the smears showed that 2 and 3 days after treatment only 27 and 38% respectively were normal. Abnormalities such as chromosomes unattached to the mitotic spindle and varying degrees of fragmentation were seen.

In order to see in greater detail the extent of chromosome damage, karyotypic analyses were made on 4 tumours at each time interval after CP and 50 chromosome spreads scored for the presense of chromatid breaks and abnormal reunion figures associated with damage by this alkylating agent; these spreads could not show abnormalities such as failure to attach to the spindle. It was observed (Fig. 2(b)) that though the peak frequency for abnormal mitotic figures occurred one day after treatment at a time when the mitotic index was still low, abnormalities could still be detected in 40% of the chromosome spreads made on the second day after treatment when the mitotic index had reached its peak value. One week after treatment only 8% of the tumour mitoses were considered to be abnormal. In control tumours from untreated rats, fewer than 1% of chromosome spreads showed comparable abnormalities.

Examination of the distribution of chromosome number in 4 tumours which had been transplanted after treatment with cyclophosphamide and had regrown at the control rates showed that a small change in the modal chromosome number had occurred. Fig. 3 shows the wide distribution of chromosome number in the untreated tumours about the mode of 65·5 (SE ± 1·6) and the distribution of chromosome number of one of the CP treated tumours which had regrown after transplantation (Mode 62·9 SE ± 0·4).

*$^3$H-TdR labelling studies*

As a further indication of the proliferative activity of the tumour following treatment with CP the $^3$H-TdR labelling indices in the tumours 0·5, 1, 2, 3 and 7 days after treatment with CP (100 mg/kg body weight) and matched controls were examined (Fig. 2(c)). The only significant difference between the control and treated animals was the increase of labelling index 12 hours after treatment.

In order to examine the distribution of tumour cells within the cell cycle 0·5, 1, 2, 3 and 7 days after CP and in the appropriate controls the DNA content of 100 cells per tumour was measured. Smears from 2 control and usually 3 experimental tumours were examined at each time interval. After measuring the DNA content the cells labelled with $^3$H-TdR were identified by autoradiography and the results are represented in Fig. 4. The DNA content of small lymphocytes in each tumour smear was used to establish the 2C mode. All tumours contained connective tissue cells with DNA content about the 2C mode. In control tumours 0·5, 1 and 2 days after T size, there was a clear cut $G_1$ mode with a DNA content of approximately 4C. There were a few cells labelled with $^3$H-TdR with DNA content between the 4 and 8C values and cells with $G_2$

FIG. 3.—Histogram of the distribution of chromosome number in (a) an untreated $RIB_5$ tumour; (b) a CP treated tumour which had regrown after transplantation to an untreated host.

DNA content were infrequent (Fig. 4(a)). Control tumours, 3 and 7 days after T size showed a slight increase in the number of cells with $G_2$ DNA content (Fig. 4(b)).

Twelve hours after CP, although there was variation amongst the 3 tumours studied, there was a decrease in the number of cells in the $G_1$ mode and a build-up of cells with higher DNA content, the majority of which were labelled with $^3$H-TdR (Fig. 4(c)). Twelve hours later (Fig. 4(d)) this build up of cells with higher DNA values had continued so that the $G_2$ mode was more pronounced than the $G_1$ mode; a moderate proportion of the cells with high DNA values were labelled. Two days after CP (Fig. 4(e)), the number of cells at the $G_2$ mode was still higher than in controls (Fig. 4(a)) but the pattern of labelling appeared to be normal. Fig. 4(f) shows that 3 days after the drug the $G_1$ peak had returned but was not as prominent as in the appropriate controls (Fig. 4(b)) and the build up of cells in $G_2$, evident 1 and 2 days after CP, was no longer so marked. Seven days after CP the normal distribution of cells was restored (Fig. 4(g)).

### DISCUSSION

Cyclophosphamide in doses 10, 50 and 100 mg/kg retarded the growth of the $RIB_5$ fibrosarcoma. In this series of experiments maximum delay of tumour growth was produced by a dose of 200 mg/kg in females, but this dose was

FIG. 4.—Histogram of the DNA content of tumour cells labelled (■) and unlabelled (□) with $^{3}$H-TdR in control tumours and in tumours 0·5, 1, 2, 3 and 7 days after 100 mg/kg CP. (a) Control tumour 0·5 days after T size; (b) control tumour 3 days after T size; (c), (d), (e), (f) and (g) represent tumours 0·5, 1, 2, 3 and 7 days after 100 mg/kg CP respectively.

lethal to tumour bearing males though males without tumours were able to tolerate this dose level. Cyclophosphamide did not make the tumours decrease in diameter. This contrasts with Thomlinson's observation (Thomlinson, 1960) that a single dose of 4000 rad of x-rays given to the tumours of rats breathing air produced tumour regression, but in these circumstances tumour growth rate was slower than we observed after cyclophosphamide treatment.

As the rate of tumour growth after treatment was not uniform regrowth has been divided into two arbitrary periods of 10 to 20 mm and 20 to 30 mm in diameter in a way similar to Thomlinson's analysis of this tumour's response to irradiation. The time taken for tumour growth from 20–30 mm after 10 mg/kg CP was the same as control tumours, but after higher doses of CP, growth from 20–30 mm in diameter was significantly delayed. This slow growth rate was probably due to the effects of CP on the tumour micro-architecture and to its residual systemic effects because when CP treated tumours were transplanted to untreated rats, tumour growth was the same as control growth even though the CP treated tumours showed a slight shift in modal chromosome number.

Measurements of gross size provide limited information about a tumour because of the many factors involved and more detailed information on the proliferative state of the tumour has been sought. These investigations have been done on smears made from minced tumour. The advantages of analysing smear preparations are the relative rapidity of the study and the fact that pooling the cells from a slice of the tumour overcomes the sampling problems encountered in sections of heterogeneous specimens (Simpson-Herren, Blow and Brown, 1968; Mendelsohn and Shackney, 1970).

Following a single dose of 100 mg/kg of CP the cytokinetic disturbances observed can be described as follows: by 12 hours after treatment, the $^3$H-TdR labelling index was almost doubled (Fig. 2(c)), and was associated with a decrease in the flow of cells into mitosis, as shown by the low mitotic index at this time (Fig. 2(a)). One day after CP the mitotic index had remained low and the DNA distribution analyses (Fig. 4(d)), showed that this was accompanied by a build up of cells in $G_2$ and by the return of the $^3$H-TdR labelling index to within normal limits (Fig. 2(c)). Twenty-four hours later the mitotic index had risen rapidly despite the build-up of cells still evident in $G_2$ and, probably as a result, $G_1$ cells were increasing in number, as shown by the return of the $G_1$ mode (Fig. 4(e)). Three days after CP the mitotic index of the tumour was still raised but fewer of these mitoses were abnormal than one and 2 days previously (Fig. 2(b)). The DNA study showed that at this time the build up of cells in $G_2$ was no longer marked. By 7 days after CP all the parameters investigated were normal.

Alkylating agents have been observed to depress mitotic activity rapidly (Layde and Baserga, 1964; Fox and Fox, 1967; DeCosse and Gelfant, 1970). Of these authors only Fox and Fox noted that the initial depression was followed, in their case of P388 cells treated *in vitro* with methyl methanesulphonate, by an increase in mitotic activity to 210% of the control values. In our investigation the high mitotic activity was probably not contributing effectively to tumour repopulation because detailed examination of chromosome spreads, one and 2 days after treatment, showed that many had abnormal chromosomes. This wave of abnormality disappeared by the end of the first week after treatment. The majority of chromosome spreads seen 12 hours after CP were normal. This observation fits well with the fact that the cell cycle of this tumour is $13{\cdot}2 \pm 1{\cdot}0$ hours (Denekamp, 1970) and that the damage to the DNA produced by alkylating agents such as CP is only visible at the second division after alkylation (Loveless, 1966).

The reason for the high $^3$H-TdR labelling index 12 hours after treatment is of some interest. This may well be due to the fact that the S period has been prolonged, although Young and DeVita (1970) suggested that CP, unlike 1,3-bis(2-chloroethyl)-1-nitrosourea, did not prolong the S period of L1210 leukaemia cells growing in an ascitic form. In our own experiments the labelling intensity of cells at 12 hours after CP was not less than that in the controls suggesting that the rate of DNA synthesis was not appreciably slower.

The DNA measurements (Fig. 4(c)) show that the majority of the labelled cells do have a DNA content above the $G_1$ value but the technique does not show whether the DNA synthesis is for repair or semi-conservative replication. The time scale of repair after alkylating agents is probably different from that after irradiation (Painter, 1970; Fox and Ayad, 1971) and repair may not be complete by 12 hours. Repair synthesis has been demonstrated by autoradiography (Fox and Ayad, 1971) but these workers used hydroxyurea to block semi-conservative DNA synthesis before detecting repair synthesis. It is not known whether cells can repair DNA during all parts of the cell cycle or whether this is restricted to the S phase (Painter, 1970; Fox and Ayad, 1971). An alternative explanation could be that there was a recruitment of cells into S from an " out of cycle " population. However, as the majority of the progeny of these cells had grossly abnormal chromosomes 12 hours later it is unlikely that many of them would contribute to the subsequent growth of the tumour.

Kinetic analyses to determine cell cycle time such as the percentage labelled mitoses method of Quastler and Sherman (1959) are not valid in the perturbed state that exists in a tumour in the first few days after treatment of the host with a cytotoxic drug. The method can be adapted to follow the flow of cells from $G_1$ and $G_2$ to mitosis in the initial hours after treatment. Estimates of the flow of cells into mitosis at later times after treatment are difficult to obtain; *e.g.* the colchicine accumulation technique is not reliable when cells have been damaged by the treatment (unpublished observation on accumulation of mitoses after CP). There are therefore limitations in the methods one can use to estimate the length of time cells spend in transit through the phases of the cell cycle.

An indication of the length of time cells spend in $G_1$, S and $G_2$ can be obtained from the relative proportions of cells in these phases. In a control tumour (Fig. 4(a)), the $G_2$ phase appeared short in comparison with S and $G_1$. This contrasted with tumours one and 2 days after CP (Fig. 4 (d), (e)), where the length of time cells spent in $G_2$ appeared to have increased.

Interpreting DNA histogram data in this way is not always straightforward. It has been discussed previously that the increase in the number of cells apparently in S 12 hours after CP (Fig. 4(c)), does not necessarily mean that the S period has been lengthened. Control $RIB_5$ tumours at T size have an appreciable out of cycle population and the most accurate estimate of the size of the growth fraction of the tumour has been suggested to be 45% (Denekamp, 1970). This means that the proportion of time cells spend in the phases of the cell cycle is only proportional to the number of cells detected in those phases by their DNA contents if one subtracts the " out of cycle " cells from the total number of cells. In control $RIB_5$ tumours we have shown (unpublished observation) that the out of cycle cells have $G_1$ DNA contents but the fate and the DNA content of the " out of cycle " cell after CP treatment is unknown. This means that accurate estimates of the length of the $G_1$, S and $G_2$ phases in the CP treated tumours from the proportions of cells in these phases are not possible. Nevertheless the marked changes in the distribution of DNA content after CP indicates the disturbances in the cellular kinetics as a result of the drug and one can observe when these disturbances are resolved.

It has often been stated (*e.g.* in *Symposium on a Critical Evaluation of Cancer Chemotherapy*, 1969) that a better understanding of the kinetics of tumour populations is necessary for more effective chemotherapy. One purpose of these experiments was to discover whether knowledge of the reaction of a tumour to cyclophosphamide could be used as a basis for the design of a rational multiple dose therapy. Dose schedules which use CP to cure certain animal tumours have been devised (Griswold *et al.*, 1968; Laster *et al.*, 1969; Teller, Bowie and Stock, 1970). In man CP is probably the most widely used alkylating agent in the treatment of late breast cancer where the clinical problems involved in finding optimal dose schedules are considerable (Stoll, 1970).

In our experiments tumour growth was compared in rats given 100 mg/kg body weight of CP in a single dose and two 50 mg/kg body weight doses separated by one, 2, 3 or 7 days. Dividing the dose did not delay overall tumour growth (10–30 mm); initial growth from 10–20 mm in diameter was faster than in rats given a single dose of 100 mg/kg but this was associated with slower growth from 20–30 mm in diameter. Varying the time of the second dose of CP was part of an attempt to compare tumour growth rate with the disturbances in the tumour cell population at the time of the second dose. However, Table I shows that despite the differences in the tumour cell populations one, 2 and 3 days after a dose of CP there were no significant differences in tumour growth rate when the second dose was given at these times.

It has been shown how the pattern of proliferative behaviour of the tumour changes rapidly as the result of treatment with an alkylating agent and biopsies at a single point of time from a treated tumour could, in the absence of information relating to the whole sequence of change, lead to misinterpretations of what was really happening. Although normal mitoses were seen 2 days after CP the distribution of cells within the phases of the cell cycle, as shown by DNA measurements (Fig. 4) had not returned to normal by 3 days. One does not know if these cells with normal mitoses are clonogenic nor is an assay available to test their clonogenicity *in vitro* or on transplantation. It is, however, cell survival *in situ* which is important in therapy where tumour blood supply, immune mechanisms, availability of metabolites and accumulation of waste products are all involved in cell survival.

In order to try to assess the nature of the overall change *in situ* morphometric studies have been made analysing the relationship of the malignant cells to the other parts of the tumour from control and CP treated animals and these results will be reported in a sequel to this paper.

Our thanks are due to Professor E. H. Cooper for his helpful discussions and to Roger Boyes, Carol Nutman and Janis Davison for their technical assistance at different times during this investigation.

This work was supported by the Yorkshire Council of the Cancer Research Campaign.

### REFERENCES

Barendsen, G. W. & Broerse, J. J. (1969) Experimental Radiotherapy of a Rat Rhabdomyosarcoma with 15 MeV Neutrons and 300 kV X-rays. I. Effects of Single Exposures. *Eur. J. Cancer*, **5**, 373.

Barendsen, G. W. & Broerse, J. J. (1970) Experimental Radiotherapy of a Rat Rhabdomyosarcoma with 15 MeV Neutrons and 300 kV X-rays. II. Effects of Fractionated Treatments, Applied Five Times a Week for Several Weeks. *Eur. J. Cancer*, **6**, 89.

Cooper, E. H., Frank, G. L. & Wright, D. H. (1966) Cell Proliferation in Burkitt Tumours. *Eur. J. Cancer*, **2**, 377.

DeCosse, J. J. & Gelfant, S. (1970) Effects of Nitrogen Mustard During the Cell Cycle of the Ehrlich Ascites Tumor. *Expl Cell Res.*, **60**, 185.

Denekamp, J. (1970) The Cellular Proliferation Kinetics of Animal Tumours. *Cancer Res.*, **30**, 393.

Fox, M. & Fox, B. W. (1967) Effect of Methyl Methanesulphonate on the Growth of P388 Lymphoma Cells and on their Rate of Progress Through the Cycle. *Cancer Res.*, **27**, 1805.

Fox, M. & Ayad, S. R. (1971) Characteristics of Repair Synthesis in P388 Cells Treated with Methyl Methanesulphonate. *Chem.-Biol. Interactions*, **3**, 193.

Griswold, D. P., Schabel, F. M., Wilcox, W. S., Simpson-Herren, L. & Skipper, H. E. (1968) Success and Failure in the Treatment of Solid Tumours. I. The Effects of Cyclophosphamide on Primary and Metastatic Plasmocytoma in the Hamster. *Cancer Chemother. Rep.*, **52**, 345.

Laster, W. R., Mayo, J. G., Simpson-Herren, L., Griswold, D. P., Lloyd, H. H., Schabel, F. M. & Skipper, H. E. (1969) Success and Failure in the Treatment of Solid Tumours. II. Kinetic Parameters and "Cell Cure" of Moderately Advanced Carcinoma 755. *Cancer Chemother. Rep.*, **53**, 169.

Layde, J. P. & Baserga, R. (1964) The Effect of Nitrogen Mustard on the Life Cycle of Ehrlich Ascites Tumor Cells *in vivo*. *Br. J. Cancer*, **18**, 150.

Loveless, A. (1966) In *Genetic and Allied Effects of Alkylating Agents*. London: Butterworths, p. 38.

Mendelsohn, M. L. & Shackney, S. E. (1970) The Growth Kinetics of Solid Tumors: Summary Report of Meeting. *Cell Tiss. Kinet.*, **3**, 405.

Moorhead, P. S., Nowell, P. C., Mellman, W. J., Battips, D. M. & Hungerford, D. A. (1960) Chromosome Preparation of Leukocytes Cultured from Peripheral Blood. *Expl Cell Res.*, **20**, 613.

Painter, R. B. (1970) Repair of DNA in Mammalian Cells. *Curr. Top. Radiat. Res.*, **7**, 45.

Quastler, H. & Sherman, F. G. (1959) Cell Population Kinetics in the Intestinal Epithelium of the Mouse. *Expl Cell Res.*, **17**, 420.

Rheinhold, H. S. (1965) A Cell Dispersion Technique for use in Quantitative Transplantation Studies with Solid Tumours. *Eur. J. Cancer*, **1**, 67.

Simpson-Herren, L., Blow, J. G. & Brown, P. H. (1968) The Mitotic Cycle of Sarcoma 180. *Cancer Res.*, **28**, 724.

Skipper, H. E. (1965) Experimental Evaluation of Potential Anticancer Agents. *Cancer Chemother. Rep.*, **45**, 5.

Skipper, H. E. (1967) Kinetic Considerations Associated with Therapy of Solid Tumours. In *21st Symp. Fundamental Cancer Res. Proliferation and Spread of Neoplastic Cells.* Baltimore: Williams & Wilkins. p. 213.

Stoll, B. A. (1970) Evaluation of Cyclophosphamide Dosage Schedules in Breast Cancer. *Br. J. Cancer*, **24**, 475.

Suntyeff, V. & Luse, S. A. (1970) Response of a Transplantable Rhabdomyosarcoma to Cyclophosphamide Therapy with Special Reference to Differences in Male and Female Mice. *Cancer, N.Y.*, **25**, 728.

Symposium (1969) A Critical Evaluation of Cancer Chemotherapy. *Cancer Res.*, **29**, 2255.

Teller, M. N., Bowie, M. & Stock, C. C. (1970) Plasma Cell Tumors in Experimental Chemotherapy: Consequences of Dose Variation on Host Survival and Paraprotein Synthesis. *J. natn. Cancer Inst.*, **45**, 1197.

Thomlinson, R. H. (1960) An Experimental Method for Comparing Treatments of Intact Malignant Tumours in Animals and its Application to the use of Oxygen in Radiotherapy. *Br. J. Cancer*, **14**, 555.

Thomlinson, R. H. & Craddock, E. A. (1967) The Gross Response of an Experimental Tumour to Single Doses of X-rays. *Br. J. Cancer*, **21**, 108.

van Putten, L. M. & Lelieveld, P. (1970) Factors Determining Cell Killing by Chemotherapeutic Agents *in vivo*—I. Cyclophosphamide. *Eur. J. Cancer*, **6**, 313.

Vrba, M. (1967) Wirkung von Endoxan auf die Chromosomen von HeLa-Zellen. *Hum. Genet.*, **4**, 362.

Young, R. C. & DeVita, V. T. (1970) The Effect of Chemotherapy on the Growth Characteristics and Cellular Kinetics of Leukemia L1210. *Cancer Res.*, **30**, 1789.